# Development and assessment of an evidence-based prostate cancer intervention programme for black men: the W.O.R.D. on prostate cancer video

**DOI:** 10.3332/ecancer.2014.460

**Published:** 2014-08-28

**Authors:** Folakemi Odedina, Awoyemi O Oluwayemisi, Shannon Pressey, Samuel Gaddy, Eva Egensteiner, Ezekiel O Ojewale, Olivia Myra Moline, Chloe Marie Martin

**Affiliations:** 1 University of Florida, Gainesville, FL 32610, USA; 2 Florida Prostate Cancer Health Disparity Research Group, University of Florida, P.O. Box 100496, Gainesville, FL 32610, USA; 3 Alachua County Prostate Cancer Alliance, Gainesville, FL 32608, USA; 4 Egensteiner Media Consulting, LLC, Gainesville, FL 32605, USA; 5 Florida A&M University, 1520 Martin Luther King Jr Blvd, Tallahassee, FL 32307, USA; 6 Howard University, 2400 Sixth St NW, Washington, DC 20059, USA

**Keywords:** prostate cancer, black men, education intervention, prostate cancer video, prostate cancer disparity

## Abstract

In spite of the numerous prostate cancer (CaP) intervention programmes that have been implemented to address the disparities experienced by black men, CaP prevention, risk reduction, and early detection behaviours remain low among black men. The lack of formal theoretical frameworks to guide the development and implementation of interventions has been recognised as one of the primary reasons for the failure of health interventions. Members of the Florida Prostate Cancer Health Disparity (CaPHD) group employed the Personal Model of Prostate Cancer Disparity (PIPCaD) model and the Health Communication Process Model to plan, implement, and evaluate an intervention programme, the ‘Working through Outreach to Reduce Disparity (W.O.R.D. on Prostate Cancer)’ video for black men. The location for the video was in a barbershop, a popular setting for the targeted group. The video starred CaP survivors, CaP advocates, a radio personality, and barbers. In addition, remarks were provided by a CaP scientist, a urologist, a CaP advocate, a former legislator, and a minister.

The W.O.R.D. video was developed to assist black men in meeting the Healthy People 2020 goal for the United States of America. The efficacy of the W.O.R.D. video was successfully established among 143 black men in Florida. Exposure to the video was found to statistically increase CaP knowledge and intention to participate in CaP screening. Furthermore, exposure to the video statistically decreased participants’ perception of the number of factors contributing to decision, uncertainty about CaP screening. Participants were highly satisfied with the video content and rated the quality of the video to be very good. Participants also rated the video as credible, informative, useful, relevant, understandable, not too time consuming, clear, and interesting.

## Introduction

The American Cancer Society (ACS) estimated that there will be about 233,000 new cases of prostate cancer (CaP) in the United States (US) in 2014, and about 29,480 men will die from the disease [1]. While there has been a decline in CaP mortality and morbidity since the 1990s, black men in the US are still disproportionately affected by CaP. In 2013, CaP accounted for 37% of all cancers diagnosed in black men [2]. The CaP incidence rate for black men was 70% higher than that of white men, while the CaP death rate was more than twice as high in black men compared to white men in 2013 [1]. Between 2005 and 2009, the average annual CaP incidence rate among black men was 63% higher compared to white men. The disproportionate burden of CaP seen in black men can be attributed to personal, provider, institutional, or health systems factors [3]. The modification of personal factors, through behavioural intervention programmes, is one of the key strategies to eliminate the CaP health disparity experienced by black men.

The personal factors of black men that have been linked to CaP disparity include low CaP knowledge [4–7], low CaP risk awareness, [8–10], and late CaP detection activities [11, 12] To address these personal factors, numerous community-based intervention programmes have been developed to enhance black men’s CaP awareness, knowledge, and early behaviours. [7, 13–16] Although some of these intervention programmes demonstrated effectiveness during the active phase of implementation, the successes have been short term. According to Green and Glasgow, most health interventions fail in targeted communities because they are impractical [17, 18]. Another reason for the failure of intervention programmes is that most are not guided by comprehensive, valid, and culturally appropriate behavioural theories. The lack of theory-based interventions greatly impacts the sustainability of prostate health intervention programmes for black men. For example, published studies continue to show that black men have limited CaP knowledge [4–7], especially with respect to personal risk assessment, and report low CaP screening [6, 19, 20].

**Figure 1.**
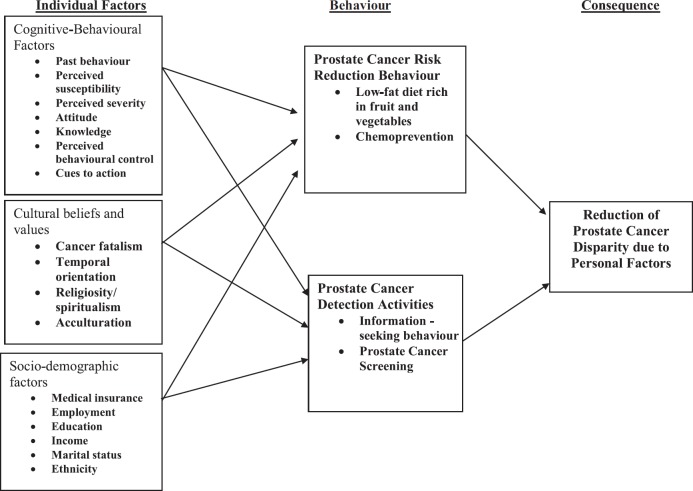
An integrative personal model of prostate Cancer disparity (PIPCaD Model) for African American men.

Based on over 10 years of research, the Florida Prostate Cancer Health Disparity Research (CaPHDR) group developed a Personal Integrative Model of Prostate Cancer Disparity (PIPCaD) for black men [4–6, 19–23]. The PIPCaD model (see Figure 1) hypothesises that a potential source of CaP disparity in black men is their personal behavioural factors such as lack of CaP risk reduction behaviours, and early CaP detection behaviours. The risk reduction behaviours include lifestyle factors such as healthy eating and exercise. Early detection behaviours include informed screening decision. The personal factors are in turn impacted by cognitive-behavioural factors (such as knowledge and health beliefs), cultural beliefs and values, and socio-demographic factors. Our central hypothesis is that an intervention programme based on a theoretical framework that predicts black men’s behaviours will succeed in improving CaP risk reduction and early detection behaviours among black men. Based on this central hypothesis, we developed the ‘W.O.R.D on Prostate Cancer Video’ (https://www.youtube.com/watch?v=74NPk6u_wcw), a prostate cancer education video, designed to close the CaP disparities gap between black men and white men.

In line with our long-term goal of improving prostate health in minority, rural, and under-served communities, the W.O.R.D video focuses on explaining the risk factors for CaP, how to reduce the risk for CaP, and informed decision making about CaP screening. The W.O.R.D video addressed the limitations of previous CaP intervention programmes for black men using two innovative strategies. The first strategy was the use of a theoretical framework, the PIPCaD model, to plan and develop a video. The PIPCaD model provided information on the behavioural factors to target the CaP education intervention, including: attitude, perceived severity, perceived susceptibility, cues to action, knowledge, and perceived behavioural control. The CaP screening facilitators and deterrents targeted are summarised in Table 1. Based on the PIPCaD model, the W.O.R.D video was developed to promote CaP risk reduction and early detection behaviours among black men by: (i) increasing awareness and knowledge about CaP and CaP prevention; (ii) supporting informed decision making about screening; (iii) promoting positive attitudes, beliefs, and practices relative to CaP prevention; and (iv) addressing barriers and facilitators to prostate screening and prostate health. Thus, the W.O.R.D video has the potential to change behaviour.

The second strategy is the use of the Health Communication Process Model [24] to ensure the successful development of the programme. The intervention is based on the six stages of continuous process of planning and improvement proposed by the Health Communication Process Model. The stages are: (1) planning and strategy selection, (2) selecting channels and materials for the intervention, (3) developing materials and pre-testing, (4) implementation of intervention, (5) assessing effectiveness of intervention, and (6) feedback to refine the programme. This study focused on the development and testing of the efficacy of the W.O.R.D video (Stage 3).

## Study objective

The primary goal of this study was to develop a video intervention that will improve the prostate health behaviour of black men. To accomplish this goal, the study objectives were to:

1. Develop a culturally relevant CaP education intervention, the W.O.R.D video, for black men based on the PIPCaD model.
2. Evaluate participants’ general assessment of the W.O.R.D video, including satisfaction with and perceived quality of the video.
3. Establish the efficacy of the W.O.R.D video in improving users’ CaP knowledge and CaP screening intention as well as reducing decisional conflict about CaP screening.

## Methods

### Study intervention: The W.O.R.D on prostate cancer video

The intervention programme was based on the PIPCaD model, which provided the understanding on the needs and perceptions of black men. The strategy statement for the intervention programme (see Table 2) was developed from the PIPCaD variables. Based on the strategy statement, the intervention includes: (1) reliable communication messages that provides culturally appropriate information on CaP for black men; (2) an efficient communication channel, a video, to facilitate the personal delivery of the interventions, increase and sustain knowledge, and promote CaP risk reduction behaviours as well as regular doctor’s visit for informed decision on early detection behaviours; and (3) trustworthy communication sources, including CaP survivors, a physician, a scientist, and a minister.

#### The intervention channel

The primary intervention channel for the W.O.R.D programme is the video. Video has been found to be one of the most effective channels for educating black men [25, 26]. It has also been used as a critical medium to promote diverse health issues. In a review of 175 studies (mostly conducted in the US), Eiser and Eiser [27] concluded that video is an effective communication tool for health interventions if it is grounded in behavioural theory and engages the audience. These authors also note that: (1) video can change attitudes and knowledge, (2) video can change behaviour if ‘actors’ are used to model the behaviour, and (3) video has been documented to increase participation in cancer screening programmes among minority women. The setting for the W.O.R.D video was a barbershop. The barbershop was selected for the setting because it is accessed by black men of diverse educational backgrounds and socio-economic classes [28–30]. Popularly regarded as ‘black men’s country club’, the barbershop has become a noted place to offer health interventions for hard-to-reach black men [13, 30–33].

#### The intervention sources

The intervention sources were primarily characters that black men can relate to and included: (1) Malik, the barber, a young black man; (2) Mr King, a 62-year-old customer, on his way to get his first prostate check-up; (3) Andre, an older customer, who has been screened regularly for years; (4) Quincy, a mid-40s customer with a comedic personality; (5) Chuck, a CaP survivor; and (6) set extras who were barbers and young customers.

#### The intervention message

The modifiable variables targeted for the intervention message were: (1) perceived behavioural control; (2) perceived susceptibility to CaP; (3) perceived severity of CaP; and (4) cues to action. A video and multimedia company was hired to produce and direct the video, including pre-production meetings and correspondence with the research team, which included CaP survivors; scriptwriting; video production planning; video shoot of all scenes; and post-production, including editing into final video sequences for website and Digital Video Disc (DVD). The storyline for the script was that Quincy (a black man in his mid-40s) becomes aware of the importance of CaP prevention and having a consultation with a physician for informed decision on screening, and finally decides to discuss it with his doctor after a lively conversation with his friends at a barbershop. A knowledgeable friend and a CaP survivor share key information with Quincy, the barbers, and other customers. While the storyline was anchored on humor, it highlighted specific messages on risk reduction and early detection of CaP. At the end of the video, the CaP message was reinforced by scrolling on the screen and simultaneously announcing information about the CaP risk factors, specific steps that can be taken to reduce an individual’s risk for cancer, the pros and cons of CaP screening, and how to make an informed decision about CaP screening.

## Study design, variables and measures

The study was a pre-, post-test research design, exploring the impact of the W.O.R.D video on the following variables: CaP knowledge, CaP screening intention, and decisional conflict. These three variables were selected as the study variables because they have been found to impact CaP risk reduction and screening behaviours [4–10]. Table 3 provides the definition of the study variables. The CaP knowledge, CaP screening intention and demographic information scales were developed and validated in previous studies by the Florida CaPHDR group [4–6, 19–23]. The measure of decisional conflict was adapted from the scale developed by O’connor [34]. In addition to the study variables, measures of satisfaction with and quality of the video were assessed after exposure to the intervention. The demographic information of participants and information on access to medical services were also assessed.

## Study setting and participants

The study setting for this project was Alachua and Orange counties in Florida. The inclusion criteria were black men, age 35 and above, regardless of CaP history. Men who were not black men of African ancestry and who were unable to speak English were excluded from participating in the study. Participants were recruited at health forums and community outreach events, including the African American Men’s Summit in Orlando, the Men’s Health Expo in Gainesville, and the University of Florida mobile health clinics in Gainesville. Participants were recruited by undergraduate research assistants, including the University of Florida-Florida A&M University ReTOOL students as part of their CaP research training experience. The research assistants approached black men at these events and asked if they would like more information about the study or have any questions about participating in the study. Only the men who indicated an interest in participating in the study were included after verbal consent.

## Data collection

Institutional Review Board (IRB) approval for the project was received on 29 March 2012. Subsequently, data were collected prospectively from participants between 1 April 2012 and 16 June 2012. Data collection included the following three steps:

**Step 1:** Participants completed a self-administered pre-test survey measuring CaP knowledge, CaP screening intention, decisional conflict, medical access information, and demographic information.

**Step 2:** Participants viewed the W.O.R.D video. The video was viewed in groups of 5–10 depending on the study site.

**Step 3:** Following the viewing of the W.O.R.D video, participants completed a self-administered post-test survey measuring CaP knowledge, CaP screening intention, decisional conflict, satisfaction with the W.O.R.D video, perceived quality of the W.O.R.D video, and general assessment of the W.O.R.D video.

The survey instructions noted that participants may choose not to answer all of the survey questions and they may withdraw from the study at any time. During data collection, participants who asked for assistance in completing the survey, mostly due to difficulty in reading, were assigned a research assistant to assist them in completing the survey. A US$10–15 Wal-Mart gift card was provided to all participants as an incentive for their participation.

## Data analysis

The survey data were coded and stored in a Microsoft (MS) Excel database. Subsequently, Psychological Clinical Science Accreditation System (PC-SAS) analytical software was employed for data cleaning and analyses. Frequency analysis was first conducted to confirm that the responses were appropriately entered and to correct any errors. The statistical analyses included: internal consistency of the study scales to establish the reliability of the measures; descriptive statistics to summarise socio-demographic, and study variables; and T-test analyses to check the changes in key study variables between pre- and post-intervention exposure.

## Results and discussion

Our primary goal was to develop an education intervention programme that will improve the prostate health behaviour of black men and establish the efficacy of the programme. Based on the Health Communication Process [24], we accomplished this goal by answering the following questions: (1) What should black men be told? (2) How do black men react to the message concepts for the W.O.R.D. video? (3) Do black men understand the message of the W.O.R.D. video, accept its importance, and agree with the value of the solution? (4) How do black men respond to the message format? (5) Based on responses from the black men, do changes need to be made in the message or in its format?

**Figure 2.**
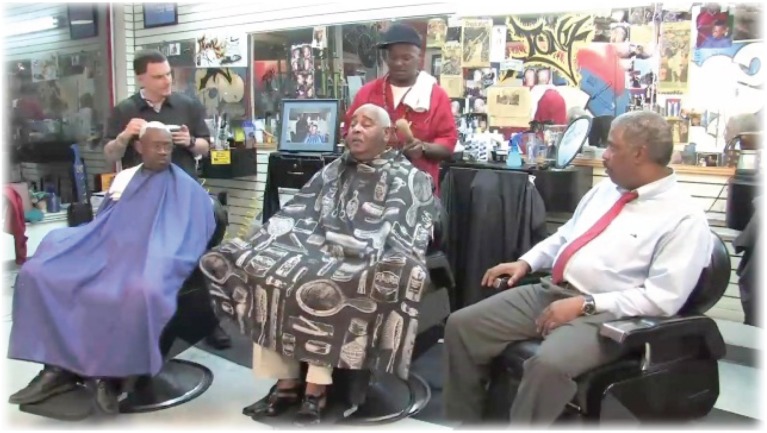
W.O.R.D. Video Scene.

The final version of the W.O.R.D video was produced on 28 February 2012. Two versions of the video were produced. The full version is about 25 minutes long and the short version is about 14 minutes long. The short version is hosted on YouTube (see http://www.youtube.com/watch?v=74NPk6u_wcw). Figure 2 is a pictorial summary of one of the scenes from the video. A total of 142 participants were recruited to establish the efficacy of the W.O.R.D video. Based on a statistical power of 0.80, a medium effect size (0.5), and probability level of 0.05 to is required to establish statistical significance, and the minimum sample size required for a two-tailed hypothesis is 128. With a sample size of 142, the study was sufficiently powered to explore the research objectives. The reliability of the study scales were: 0.76 for Intention pre-test scale; 0.70 for satisfaction scale; 0.78 for perceived quality scale; and 0.86 for intention post-test scale. All the scales were found to be reliable based on Nunnaly’s [35] suggestion of 0.70 to be an acceptable reliability coefficient.

Table 4 provides a summary of the characteristics of study participants. The study participants were mostly US-born black men, between 50 and 59 years, married, urban residents, who had full-time employment, with an education level of high school diploma, and earned less than US$20,000. In addition, most of the participants had insurance and access to a primary care provider. The majority of the participants were recruited from the African American Men’s Health Summit held at the Orange County Convention Centre in Orlando. Given that the majority of the men who attend the summit have participated for many years, our expectation was that their CaP knowledge and intention to screen for CaP would be relatively high.

## Participants’ prostate cancer knowledge, intention to screen, and decisional conflict

A summary of participants’ responses on the study variables is provided in Table 5. The mean pre-test knowledge score was 63.6%, and the mean post-test knowledge score after intervention exposure was 74.0%. Table 6 provides additional details on the performance of participants on the knowledge scale. The questions that participants were least knowledgeable about (scored less than 60%) were: (1) I should be able to tell immediately if I have prostate problem; (2) Having somebody in your family with prostate cancer increases the chance of getting prostate cancer; (3) A diet high in fat will decrease the chance of getting prostate cancer; (4) Doing only one of the tests, prostate-specific antigen (PSA) or the digital rectal exam (DRE), is enough to test for prostate cancer; and (5) Early screening for prostate cancer cannot tell if one has prostate cancer. Interestingly, there was an increase in the overall performance of participants on all knowledge items after watching the W.O.R.D video, including the five areas of difficulty noted above.

The behavioural intention of participants relative to participating in CaP screening within the next year was measured by three items with a scale ranging from 3 to 15. As expected, the baseline intention of the participants was high given that the majority of the participants were recruited at a health summit. The pre-test score was 12.78 and the post-test score 13.37. While the baseline score of participants on the intention scale was high, the intervention exposure slightly increased the mean score of participants on this scale.

According to O’Connor [34], decisional conflict is a participant’s state of uncertainty about the course of action to take when making choices involving risk or uncertainty of outcomes, such as the decision to screen for CaP. The decisional conflict scale (DCS) comprises three subscales: (1) uncertainty; (2) selected factors contributing to the uncertainty (referred to as uncertainty factors); and (3) perceptions of effective decision making. The first two subscales were adapted for this study. Participants’ pre-test score on decision uncertainty was 10.44 on a scale ranging from 3 to 15. The post-test score was 10.81. A lower score on the uncertainty scale indicates high uncertainty. Thus, the uncertainty of participants decreased slightly after watching the W.O.R.D video. The second subscale for the decisional conflict scale is uncertainty factors. Seven items were used to assess this subscale, with scale score ranging from 7 to 35. A higher score on this scale indicates greater number of factors contributing to uncertainty. The pre-test score on the uncertainty factor subscale was 17.31, and the post-test score 16.42. Similar to the uncertainty subscale, the number of uncertainty factors perceived by participants decreased slightly after watching the W.O.R.D video.

## Participants’ assessment of the W.O.R.D video

In general, the assessment of the W.O.R.D. video was highly positive. Over 90% of the participants indicated that they were satisfied with the video, and 80% of the participants indicated that the quality of the video was high. The mean satisfaction rating was 13.67 on a scale ranging from 3 to 15, indicating a highly satisfactory rating for the video. The mean quality score was 12.19 on a scale ranging from 3 to 15, indicating very good rating. Table 7 provides a summary of participants’ assessment of the W.O.R.D. video. Over 75% of the participants indicated that: (1) the information provided by the video was useful to them; (2) they understood all the words used in the video; (3) they did not feel that the video was too long or too time consuming; (4) they did not find any of the information on the video embarrassing; (5) they did not experience any difficulty with the video; (6) the video was relevant to them; (7) the video got their attention; (8) the video has the potential to increase CaP knowledge among black men; and (9) they found the video to be credible. These assessments indicate the comfort level of participants with the video. This is especially important given that the majority of the participants’ educational level was high school diploma.

The use of video as an intervention channel has several advantages as a powerful communications vehicle, including the fact that [27] (1) it is vivid and graphic; (2) it enabled us to display real-life situations through actors; (3) it facilitated communication of appropriate behaviour for the audience; (4) it facilitated the provision of the information in a very lively and humorous way; (5) it fostered engagement of the audience, thereby encouraging them; (6) the actors were able to personalise the issues presented on the video; (7) as noted by the participants, the video overcame difficulties with literacy; and (8) it will continuously engage the population through social media, in this case YouTube. It is interesting to note that the participants did not indicate a preference for viewing the video in a health care setting. When asked whether they would feel more comfortable viewing the video at a health care office (such as a doctor’s office, pharmacy, or clinic), only 42% of the participants indicated yes. Our goal was to have black men feel comfortable viewing the video in any setting, including barbershops. Almost 60% of the participants indicated that they would be comfortable viewing the video outside a health care setting.

## Efficacy assessment of the W.O.R.D video

Student’s T-test analysis was conducted to compare the differences between the means of the pre-test scores and post-test scores on CaP knowledge, intention to screen for CaP, decision uncertainty and uncertain factors after the intervention exposure. A summary of the T-test results is provided in Table 6. Based on the p-values, exposure to the W.O.R.D video statistically improved CaP knowledge, increased intention to screen for CaP within the next year, and decreased participants’ perception of the number of factors contributing to uncertainty about CaP screening. Although the uncertainty of participants relative to making a decision about CaP screening decreased slightly after watching the W.O.R.D video, the decrease was not statistically significant. This may be because it may be necessary for the participants to review additional information and process the information in order to effectively impact decision uncertainty. In this case, print media would be ideal to reinforce the information on the video. Subsequently, an educational brochure based on the information presented in the W.O.R.D. video has been developed. A wallet-size screening information card has also been developed for black men to document the results of their screening tests. The card also lists the risk factors for CaP. In addition to providing chart fields to record CaP test results for five years, the card serves as a reminder to discuss CaP screening with a physician annually.

## Conclusions

Black men were the only racial group that did not meet the CaP Healthy People 2010 goal for the US to reduce the CaP death rate to 28.8 deaths per 100,000 males by 2010 [36]. Between 2005 and 2009, the CaP death rate for black men was 53.1/100,000 compared to 21.7/100,000 for white men [2]. While black men are yet to meet the Healthy People 2010 goal for CaP, the Healthy People 2020 goal target is 21.2 CaP deaths per 100,000 men [37]. For black men, the Healthy People goal may continuously be a moving target that remains unattainable unless an intervention that effectively addresses the factors that have been noted to cause CaP disparity in this at-risk group is implemented.

The W.O.R.D. video was developed to assist black men in meeting the Healthy People 2020 goal for the USA. The efficacy of the W.O.R.D. video was successfully established among 143 black men in Florida. Exposure to the W.O.R.D. video was found to statistically increase CaP knowledge and intention to participate in CaP screening. Furthermore, exposure to the W.O.R.D. video statistically decreased participants’ perception of the number of factors contributing to decision uncertainty about CaP screening. Participants were highly satisfied with the video content and rated the quality of the video to be very good. Participants also rated the video as credible, informative, useful, relevant, understandable, not too time consuming, clear, and interesting.

It is important to note that the benefits of CaP screening for the general population remain controversial. However, given the disproportionate burden of CaP in black men and the lack of clinical trials to assess the effectiveness of screening in black populations, the only strategy we currently have to combat CaP disparity for black men is the early detection of CaP. The W.O.R.D. video promotes informed decision making by black men, listing the pros and cons of CaP screening.

This project only focused on the short-term impact of the W.O.R.D. video, focusing on its efficacy and not the effectiveness in real life settings. The next step would be to assess the effectiveness and uptake of the intervention. While there are several advantages to providing CaP education at barbershops, as pointed out above, it is important to note that this cannot serve as a surrogate to talking with a healthcare provider. The W.O.R.D. video intervention is likely to be more effective in barbershops if the barbers are trained to be prostate health advisers. In the absence of trained barber health advisers, the barbers will need to navigate their clients to appropriate health care providers for prostate health education. Another limitation is the representation of participants. Since participants were not recruited randomly and constitute a convenient sample, they are not representative of all black men. As mentioned above, the participants for the study were more knowledgeable about CaP and had a higher CaP screening intention because of their exposure to CaP information at health forums. In spite of these limitations, the W.O.R.D. video has significant advantages. It was tailored to the specific needs and characteristics of black men because it was developed from information generated from black men on their CaP personal factors. The W.O.R.D. video message is thus credible, relevant, and culturally-responsive to black men. The W.O.R.D. video is also highly accessible nationwide through YouTube, fostering widespread dissemination, at minimal cost. In addition, the W.O.R.D. video DVD and accompany brochures are available at no charge to black men.

## Conflicts of interest

There is no conflict of interest reported by authors.

## Figures and Tables

**Table 1.** Prostate cancer behavioural factors addressed in the W.O.R.D video.

Prostate Cancer Screening Facilitators	Prostate Cancer Screening Deterrents
• Prostate cancer signs and symptoms	• Fear of testing positive for prostate cancer
• Recommendations by doctors	• Low perceived susceptibility to prostate cancer
• Having appropriate information	• Limited access to screening
• Access to screening	• No information provided by doctor
• Less invasive procedure	• Do not trust doctor
• Knowledge of risk factors	• No regular primary care provider
• Family encouragement	• Uncomfortable screening procedure
• Perceived benefits of screening	• Low perceived risk of developing prostate cancer
• Media campaign	• Lack of information about the disease
• Perceived seriousness of prostate cancer	
• Prostate cancer screening awareness	
• Knowing somebody who has prostate cancer	

**Table 2.** Strategy statement for the PIPCaD programme.

**Audience.** Black men, including African American, African and Caribbean men between the ages of 40 and 70 years.	
**Objectives.** To improve black men’s: (i) perceived behavioural control—confidence of participants’ ability to reduce the risk for prostate cancer and regular doctor’s visit for informed decision on early detection; (ii) perceived susceptibility to prostate cancer—opinion of chances of getting prostate cancer; (iii) perceived severity of prostate cancer—opinion about the seriousness of prostate cancer and its sequel; and (iv) cues to action from healthcare providers—strategies to inform about and activate prostate cancer risk reduction and early detection.	
**Examples of obstacles identified by black men.**	
Fear of testing positive for prostate cancer	Low perceived susceptibility to prostate cancer
Limited access to screening	No information provided by doctor
Does not trust doctor	No regular primary care provider
Uncomfortable screening procedure cancer	Low perceived risk of developing prostate
Lack of information about the disease	
**Examples of facilitating factors identified by black men.**	
Prostate cancer signs and symptoms	Recommendations by doctors
Having appropriate information	Access to screening
Less invasive procedure	Knowledge of risk factors
Family encouragement	Perceived benefits of screening
Media campaign	Perceived seriousness of prostate cancer
Prostate cancer screening awareness	Knowing somebody who has prostate cancer
**Communication sources.** Black men’s organisations; physicians; other health care providers, including community pharmacists; accessible providers like neighborhood barbers.	
**Communication content.** Prostate cancer information including prevention, disease symptoms, and early detection; addressing the myths and misunderstanding about prostate cancer; and how to ask the right questions about prostate cancer when communicating with health care providers.	
**Communication channel.** Videos, print materials, health screening guide, websites, and flyers.	
**Programme Partners.** Prostate cancer survivors; advocates; community-based organisations; Florida Department of Health; American Cancer Society; Cancer Information Service.	

**Table 3.** Table 3. Conceptual and operational definitions of study variables.

Conceptual definition	Operational definition
Prostate cancer knowledge—Participants’ understanding of CaP disease, prevention and detection.	Ten questions assessed participants’ prostate cancer knowledge using a True/False/Don’t Know scale. Each correct response had 1 point and incorrect response or ‘I don’t know’ response had 0 point. Higher score indicated high knowledge of prostate cancer information.
Prostate cancer early detection behaviour—CaP information seeking behaviour and CaP screening.	An index of Prostate Cancer Detection Behaviour was created by combining (sum) the responses of participants on prostate cancer screening by DRE in the last year (5 if Yes, and 0 if No); prostate cancer screening by PSA in the last year (5 if Yes, and 0 if No); prostate cancer information seeking behaviour (measured on a 5-point Strongly Agree–Strongly Disagree scale); prostate cancer discussion with a physician (measured on a 5-point Strongly Agree–Strongly Disagree scale); and paying attention to prostate cancer information (measured on a 5-point Strongly Agree–Strongly Disagree scale). The index score ranged from 0 to 25.
Prostate cancer screening intention—Participants’ expressed likelihood of screening for CaP in the future	Three questions were used to measure intention. For example, participants responded to the question: ‘Within the next year, I will get tested for prostate cancer with the Digital Rectal Examination (DRE)’ on a strongly disagree (1)–strongly agree (5) scale, with higher score indicating high intention.
Decisional conflict—Participants’ state of uncertainty about the course of action to take relative to CaP screening [34].	O’Connor’s scale [34] provided the items for decisional conflict. The scale used for this study assessed CaP screening uncertainty (3 items) and factors contributing to uncertainty (7 items) on a strongly disagree (1)–strongly agree (5) scale. An example of the screening uncertainty measure is ‘I’m unsure what to do when it comes to prostate cancer screening’. Lower score on this scale indicates high uncertainty. An example of the factors contributing to uncertainty measure is ‘I feel I know the risks and side effects of prostate cancer screening’. Higher score on this scale indicates greater number of factors contributing to uncertainty.
Satisfaction with the W.O.R.D video	Participants’ satisfaction was assessed by three questions. For example, participants responded to the statement, ‘The information I received from the Prostate Cancer Education video can best be described as’ using a Very Unsatisfactory (1)–Very Satisfactory scale (5) with higher score indicating high satisfaction.
Quality of the W.O.R.D video	Participants’ perceived quality was assessed by three questions. For example, participants responded to the statement, ‘The Prostate Cancer Education video can best be described as superior’ using a strongly disagree (1) strongly agree (5) scale, with higher score indicating high quality rating.
General assessment of the W.O.R.D video	Using a ‘Yes’, ‘No’ and ‘I can’t say’ scale, participants responded to ten questions focused on general assessment of the video. Examples were: Was the information provided by the video useful to you? Did you experience any difficulty with the video? In your opinion, would you say that the video is credible?

**Table 4.** Participants’ demographic and health characteristics.

Variable	Frequency	Percent (%)
**Ethnicity**		
African American of American origin	107	79%
Black Immigrants (African, Caribbean), (European, S American)	14	10%
Other	15	11%
Frequency Missing	6	N/A
**Age (years)**		
Less than 40	16	11%
40 to 49	35	25%
50 to 59	41	30%
60 to 69	31	23%
70 to 79	12	9%
80 years and above	3	2%
Frequency Missing	4	N/A
**Education**		
Less than high school	9	7%
High school degree	51	37%
Some college training	26	19%
College degree	41	30%
Post-college degree	9	7%
Frequency Missing	6	N/A
**Marital status**		
Single	35	26%
Married	77	56%
Divorced	22	16%
Widowed	3	2%
Frequency Missing	5	N/A
**Employment**		
Full time	59	44%
Part time	11	8%
Disability	14	10%
Retired	31	23%
Unemployed	21	15%
Frequency Missing	6	N/A
**Household income (US$)**		
0-19,999	41	31%
20,000–39,999	36	27%
40,000–59,999	17	12%
60,000–79,999	21	16%
80,000 – 99,999	5	4%
100,000 and above	13	10%
Frequency Missing	9	N/A
**Insurance**		
Yes	99	72%
No	39	28%
Frequency Missing	4	N/A
**Access to regular physician**		
Yes	101	73%
No	37	27%
Frequency Missing	4	N/A
**Residence**		
Rural	43	33%
Urban	86	67%
Frequency Missing	13	N/A

**Table 5.** Participants’ responses on study variables and T-Test results for pre- and post-test analyses.

Study Variables	Scale Range	Pretest Mean Score (SD)	Posttest Mean Score (SD)	*P* - value	Interpretation
Knowledge (percentage)	0–100	63.60 (22.20)	74.00 (16.80)	0.0021	Statistically Significant
Intention	3–15	12.78 (2.48)	13.37 (2.13)	<.0001	Statistically Significant
Decision uncertainty	3–15	10.44(2.57)	10.81 (2.88)	0.1001	Not Significant
Uncertainty factors	7–35	17.31 (4.42)	16.42 (4.68)	0.0170	Statistically Significant
Satisfaction	3–15	13.67 (2.01)		Not applicable	Not applicable
Video quality	3–15	12.19 (2.52)		Not applicable	Not applicable

**Table 6.** Participants’ responses on knowledge scale pre- and post-intervention exposure.

	Percent of participants with correct responses on:	
	Pre-test Scores	Post-test Scores
**1.** Prostate cancer is the most common cancer in men. (True)	81.69	92.25
**2.** I should be able to tell immediately if I have prostate problem. (False)	50.70	60.56
**3.** Black men are more likely to get and die from prostate cancer than any other men. (True)	82.39	97.18
**4.** Having somebody in your family with prostate cancer increases the chance of getting prostate cancer. (True)	57.04	76.76
**5.** Getting up often at night to pass urine may be a sign of prostate cancer. (True)	60.56	66.20
**6.** A diet high in fat will decrease the chance of getting prostate cancer. (False)	44.37	50.70
**7.** The two main tests for prostate cancer are the blood test called Prostate Specific Antigen (PSA) and the Digital Rectal Exam (DRE) where a gloved finger is placed in the rectum to feel the prostate. (True)	80.14	93.62
**8.** Doing only one of the tests, Prostate Specific Antigen (PSA) or the Digital Rectal Exam (DRE), is enough to test for prostate cancer. (False)	44.37	54.93
**9.** Early screening for prostate cancer cannot tell if one has prostate cancer. (False)	47.18	52.82
**10.** It is often suggested that black men over the age of 40 should get tested for prostate cancer every year. (True)	88.03	95.77

**Table 7.** General assessment of W.O.R.D video.

General Assessment Items	Percent Frequency (%)			
	Yes	No	I can’t say
Was the information provided by the video useful to you?	98.56	0.72	0.72
Were there any words used in the video that you did not understand?	23.18	75.36	1.45
Do you feel that the video was too long or too time consuming?	15.72	84.28	-
Did you find any of the information embarrassing?	13.48	86.52	-
Did you experience any difficulty with the video?	19.28	78.57	2.14
Was the video relevant to you?	82.98	15.60	1.42
Did the video get your attention?	93.57	4.29	2.14
In your opinion, would you say that the video has the potential to increase prostate cancer knowledge among black men?	94.33	4.26	1.42
In your opinion, would you say that the video is credible?	94.97	2.88	2.16
Would you feel more comfortable viewing the video at a health care office, such as a doctor’s office, pharmacy, or clinic?	42.14	40.71	17.14
